# Energy Storage and Dissipation in Consecutive Tensile Load-Unload Cycles of Gum Metal

**DOI:** 10.3390/ma16093288

**Published:** 2023-04-22

**Authors:** Karol Marek Golasiński, Maria Staszczak, Elżbieta Alicja Pieczyska

**Affiliations:** Institute of Fundamental Technological Research, Polish Academy of Sciences, Pawińskiego 5B, 02-106 Warsaw, Poland; kgolasin@ippt.pan.pl (K.M.G.); mstasz@ippt.pan.pl (M.S.)

**Keywords:** gum metal, β-Ti alloy, cyclic tension, superelasticity, energy balance, dissipation, stored energy, infrared thermography

## Abstract

Multifunctional β-titanium alloy Gum Metal, characterized by a relatively low elastic modulus, superelastic-like behavior and high strength, was subjected to cyclic tensile loadings. The characteristics of macroscopic scale energy storage and dissipation in the consecutive loading–unloading cycles were studied. Various kinds of energy components related to the alloy deformation process were determined experimentally and analyzed using thermodynamic relations. The values of the entire work needed to deform the alloy $W_{ext}$, the work used for recoverable deformation $W_{rec}$ consisting of the elastic deformation energy $W_{el\;}$, the superelastic-like energy $W_{pt\;}$, and the energy of thermoelastic effect $E_{th\;}$, were derived from the Gum Metal stress and temperature vs. strain curves. The irrecoverable mechanical energy $W_{ir}$ expended on plastic deformation, the dissipation energy Q, and finally the stored energy $E_{s}\;$ were estimated. The stored energy represents a change in the internal energy of the deformed material and is an essential measure of cold-worked state. The $E_{s}$ value turned out to be not large for the Gum Metal, which confirms the alloy low hardening property. The energy components determined for each of the 24 loading cycles enabled us to analyze various stages of the Gum Metal deformation process, including necking and damage.

## 1. Introduction

The study of the energy balance in the process of plastic deformation of a metal, alloy, or polymer is an important challenge since it is just energy, especially the energy transition, that determines the thermodynamic conditions responsible for the current state of the material and its instantaneous changes, including the deformation mechanisms and the resulting structure. 

Theoretical analyses of such key aspects of the materials’ behavior as an energy balance were started by Taylor and Quinney, 1934 [1], who named energy storage as “latent energy” or “latent heat”, underlining its hidden nature. However, it took time to get particular instruments to confirm the idea by any material experimental testing. Thus, the energetic aspects of the behavior of the material under loading were developed by other researchers according to the development of experimental techniques and proposed thermodynamic descriptions, e.g., Bever and Holt [2]. The backgrounds of the reversible thermodynamics of metals under stress for such analysis were presented, i.e., by Beghi, Bottani, and Caglioti in [3]. The experimental determination of the energy stored in alloys was conducted by Chrysochoos et al. [4,5,6,7,8,9]. They were the first who performed the mechanical tensile loading inside the specially modified calorimeter, captured required data (heat), and estimated the dissipated and stored energy during the deformation process of various materials. 

Gadaj et al. [10], Oliferuk et al. [11,12], and Oliferuk [13] reported energy stored related to the plastic deformation of stainless steel, based on experimentally obtained stress–strain curves and the related temperature changes. The temperature of the specimen caused by the thermomechanical couplings was captured by infrared camera. The temperature changes of the specimen due to the Joule-Lenz effect were measured in the same set-up, in an especially proposed experiment aimed at estimating the heat exchange with the surroundings during the deformation process. The same methodology was used by Pieczyska in [14] in order to estimate the energy storage in subsequent loading–unloading cycles in stainless steel. In addition, the sum of the energy obtained for the consecutive cycles was compared to that obtained from the single one, to the same strain limit. The mechanical characteristics and the related temperature data of this research were reported by Pieczyska [15,16] and Pieczyska et al. [17]. The thermomechanical analysis of the cyclic behavior of metals was presented by Chrysochoos in [18]; a valuable overview was reported in [19], whereas Digital Image Correlation (DIC) and Infrared Thermography Technique (IRT) applied for the thermomechanical analysis of material behavior were presented by Chrysochoos et al. in [20].

Energy storage and dissipation in the TiNi shape memory alloy (SMA) subjected to tension and demonstrating pseudoelastic behavior under various thermomechanical loading conditions were estimated by Pieczyska and Tobushi et al. in [21]. 

Knysh and Korkolis proposed a new experimental approach for measuring the fraction of plastic work converted into heat in metals [22]. They investigated four materials—i.e., 303 and 316 stainless steels, commercially-pure titanium, and titanium alloy applied for medical implants Ti–6Al–4V. The elaborated fractions of plastic works were not constant during the loading; the energy decreased with the accumulation of plastic deformation. 

The theoretical background used for the determination of the energy storage in solids due to plastic deformation assumed that the strain state is homogeneous. However, one is aware that, as the load increases, the deformation becomes heterogeneous, and the strain localization develops, leading to the specimen necking and damage. The distribution of energy storage rate in the area of strain localization during tension was analyzed in stainless steel by Oliferuk et al. The experimental procedure was based on the simultaneous measurements of the temperature and displacement distributions on the specimen surface. To this end, infrared thermography and visible light imaging were used [23]. A study on the topic of the conversion of plastic work to heat in textured magnesium alloy AZ31B was presented by Kingstedt and Lloyd in [24]. The experiments showed that the amount of plastic work converted into heat was a function of the strain and the predominant deformation of the alloy. Yazdandoost et al. investigated experimentally and computationally the energy dissipation of shock-induced stress waves through phase transformation and plastic deformation in TiNi SMA, considering stress-induced phase transformation [25]. It was confirmed that, in SMA, the energy dissipation was mainly induced by the reversible phase transformation; the plastic deformation had a minor contribution to the energy dissipation. Neto et al. used experimental and numerical approaches to evaluate the temperature variation observed during quasi-static uniaxial tensile tests in aluminum alloy AA6016-T4 due to the heat generated by plastic deformation [26]. It was also reported that the fraction of plastic work converted into heat increased with the accumulation of plastic deformation.

Sharkeev et al. in [27] and Legostaeva et al. in [28] studied pure titanium, Zr-1Nb, and Ti-45Nb alloys in various microstructural states using infrared thermography. Significant differences between the coarse-grained and ultrafine-grained states of the materials in terms of the regularities of energy accumulation and dissipation during plastic deformation were demonstrated.

Musiał et al. performed an experimental and theoretical micro-scale analysis of the energy conversion process during uniaxial tension of 310S steel by using IRT and DIC techniques coupled by ThermoCorr 1.0 software [29]. The energy stored and dissipated as well as the distributions of plastic work considering the plastic anisotropy under various strain rates were identified. 

The estimation of the components of the energy balance, especially the stored energy, is particularly valuable in the case of new materials demonstrating unique properties and unconventional deformation mechanisms. One of them is a β-Ti alloy, which combines high strength and elastic properties; thus, it was named Gum Metal by the inventor [30].

Gum Metal is a trademark of the Toyota Central Research and Development Laboratories, Inc. coined in 2003, which stands for a group of β-titanium alloys that exhibit multiple super properties and drastic changes in physical properties after plastic working at room temperature. These alloys simultaneously offer low Young’s modulus, high strength, excellent cold-workability, and stable thermal properties, as well as superelastic-like behavior which refers to a large range of nonlinear recoverable deformation without hysteresis. 

In the first papers published on Gum Metal, it was speculated that such a set of outstanding properties, activated in the alloy under mechanical loads, was caused by unconventional, dislocation-free deformation mechanisms—namely, giant faults—which are macroscopic planar defects carrying very large plastic strains [30,31,32,33,34,35,36]. Intensive experimental research, in particular systematic and comprehensive structural investigations, allowed for certain verification of these initial assumptions. In further studies, other features of the microstructure were also observed, e.g., nanometer-sized ω phases that contribute to the mechanisms of deformation in Gum Metal [37,38,39]. 

In the papers reported by Raabe et al. [40], it was also claimed that the plastic behavior of Gum Metal is governed by the presence of ω nanoparticles. Selected zones, significantly depleted of ω precipitates, were said to be channels that appear as widely spaced deformation bands. A considerable amount of plastic flow was found to be concentrated in the channels, which produce shear steps and large shear displacements, also called giant faults [40,41,42,43]. The role of nanoprecipitation in a β-Ti alloy was raised by Coakley et al. in [44], whereas analysis of trace oxygen for dislocation-free deformation and superelastic load cycling was reported in [45,46,47]. 

Comprehensive research on Ni-free and β-Ti alloy conducted by Miyazaki, Kim et al. at the University of Tsukuba demonstrated that the superelastic-like behavior of Gum Metal is related to the fact that the long-range phase transformation of oxygen-free Ti-Nb based alloys is hindered by the addition of oxygen. The local lattice distortion around the oxygen atoms, named a nanodomain, is an intermediate phase between the β and the α” martensite phase [48,49,50,51]. Pieczyska et al. in [52,53] and Golasiński et al. in [54] confirmed the increase of the Gum Metal temperature in this superelastic region by using IRT, related to the exothermic activity of the nanodomains. The thermomechanical behavior of Gum Metal in cyclic loading in view of unconventional deformation mechanisms was discussed in [55]. A finite strain elastic–viscoplastic model of the Gum Metal, considering cyclic loading, was presented in [56]. 

The energetic aspect of energy storage and dissipation is important for all materials subjected to deformation, and a similar approach can be applied to any solid material; e.g., various metals, alloys, and polymers. To date, the research is developed mainly for stainless steel and other materials that are easily available on the market, with well-known mechanisms of plastic deformation.

The proposed paper concerns the estimation of energy balance in Gum Metal subjected to consecutive tensile loading using a macroscopic approach. A state of art of the methodology and unique Gum Metal properties are introduced in Section 1. The experimental apparatus, material and specimens are described in Section 2. Mechanical and the related temperature changes for Gum Metal cyclic loading are shown in Section 3. The theoretical background for the estimation of energy balance is presented in Section 4. The mechanical results obtained by DIC as well as the related temperature changes captured by IRT for 24 loading–unloading cycles are depicted in Section 5. The plastic and dissipated work, as well as stored energy in Gum Metal subjected to the loading cycles are determined and discussed in Section 6. The experimental procedure, challenges, and obtained results are summarized in Section 7.

## 2. Experimental Procedure

An estimation of the macroscopic scale energy balance in Gum Metal was conducted for 24 loading–unloading tension cycles at strain rate 10^−2^ s^−1^ and the displacement step of 0.05 mm in each subsequent cycle up to the specimen rupture. After each cycle, the test was paused for 2 min in order to let the specimen return to the room temperature before the subsequent loading.

### 2.1. Material

The material used in the experiment was a Gum Metal specimen with the composition of Ti–23Nb–0.7Ta–2.0Zr–1.2O (at.%) fabricated by the Toyota Central Research & Development Laboratories Inc. using a powder process as described in [31]. A billet was sintered at 1300 °C for 16 h in a vacuum of 10^−4^ Pa, hot forged to a 15 mm round bar, solution treated at 900 °C for 30 min, and subsequently quenched into water with ice. The heat-treated bar was removed with a surface oxidized layer and cold-worked by a rolling machine down to the thickness of 0.5 mm. The specimen was cut using electrical discharge machining to obtain a gauge area of 7 mm × 4 mm.

### 2.2. Experimental Setup

**Figure 1:**
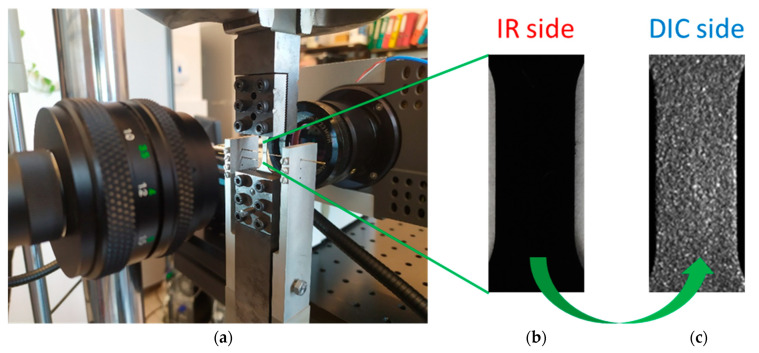
(**a**) Photograph of Gum Metal specimen in the grips of the testing machine monitored by two cameras working in visible and infrared spectra and two sides of the gauge area covered with (**b**) soot (IR side) and (**c**) a speckle pattern (DIC side).

A photograph of the experimental setup is shown in Figure 1a. It consists of MTS 858 testing machine and two cameras working in different spectral ranges. A ThermaCam Phoenix IR was working in the infrared range and a Manta G-125B camera was operating in the visible range. The wave length of the IR is 3–5 μm, the maximal recording frequency is 538 Hz, and the thermal sensitivity is up to 0.02 °C [53,54]. The cameras were placed on the opposite sides of the Gum Metal specimen. One side of the gauge area of the specimen was covered with soot (see Figure 1b) to increase and make uniform the emissivity, which was assumed to be 0.95. The other side was covered with a speckle pattern of white paint with micrometer-size metal particles in order to apply the DIC technique, as presented in Figure 1c.

From the obtained temperature distribution, the mean temperature was determined considering the internal gauge area of the specimen to neglect boundary effects. The temperature change Δ*T* denotes the difference between the mean value of the temperature calculated for the gauge area of the tested specimen at each instant of straining *T (t)* and the mean temperature of the same area before the deformation *T (t_0_)*:

(1) $$\Delta T=T\left(t\right)-T\left(t_{0}\right).$$

The mean temperature value was calculated for the gauge part of the specimen defined in the reference configuration. ThermoCorr 1.0 software with the algorithm developed at the IPPT [57] was used to correlate the obtained mechanical (DIC) and thermal (IRT) experimental data. A function of virtual extensometer was used to calculate strain and plot stress vs. strain curves.

## 3. Mechanical and the Related Temperature Changes for Gum Metal Cyclic Loading

**Figure 2:**
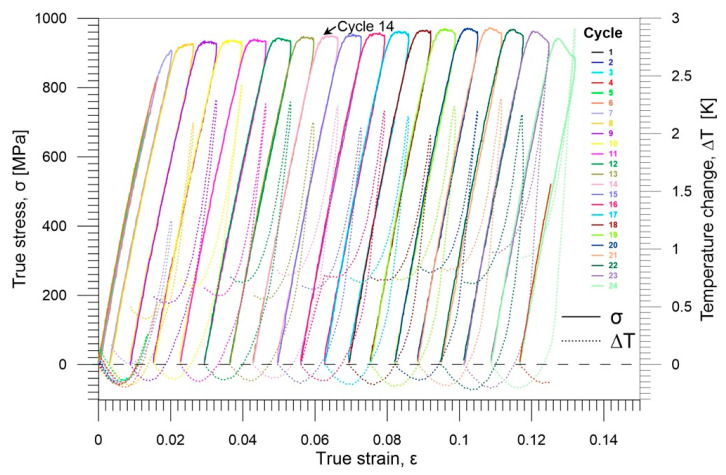
Stress *σ* accompanied by the related average temperature change Δ*T* vs. strain *ε* curves obtained for tensile loading–unloading cycles 1–24 of Gum Metal at the strain rate of 10^−2^ s^−1^.

The true stress *σ* and the related average temperature change Δ*T* vs. true strain *ε* curves, denoted by the same color for the particular loading-unloading cycle, are depicted in Figure 2. The true stress and the true strain values were calculated in reference to the current cross-section of the sample assuming its constant volume. More details are presented in [55].

Looking at the mechanical and the related thermal curves presented in Figure 2 it can be observed that due, to the thermoelastic effect, the temperature of the specimen decreases during the initial stage of any loading cycle next increases in the Gum Metal advanced superelastic region, which is followed by a significant increase, related to the plastic deformation. In general, the same tendency in thermomechanical response is observed for each of the cycles 6–24. However, it is seen that, in the initial loading and unloading cycles 1–5, the true stress vs. true strain plots overlap because the deformation occurs in the elastic, reversible regime [55,56].

The obtained results are affected by heat exchange with the surroundings. Nevertheless, in these conditions, the effect is not too significant. At the strain rate applied, i.e., 10^−2^ s^−1^, we can assume that the process is close to the adiabatic. 

**Figure 3:**
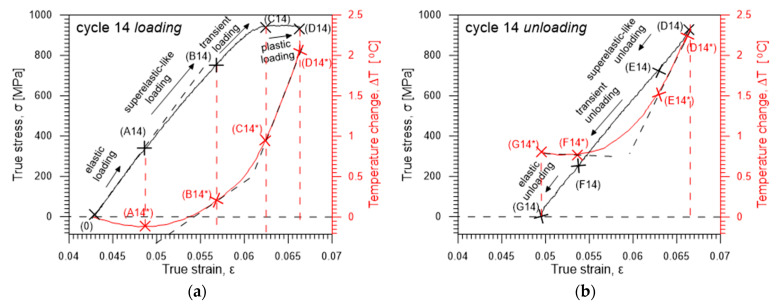
Characteristics of cycle 14 with particular stages of the Gum Metal deformation: stress *σ* (black line) accompanied by the average temperature change Δ*T* (red line) vs. strain *ε* curves during (**a**) loading, (**b**) unloading.

In order to better understand the loading–unloading process, the characteristics of an exemplary cycle 14 with an advanced deformation were analyzed in more detail. The stress *σ* accompanied by the related average temperature change Δ*T* vs. strain *ε* curves are presented for the loading and unloading in Figure 3a,b, respectively. The curves demonstrate that apparent plastic deformation in Gum Metal takes place.

Specific stages of Gum Metal deformation during loading and unloading, identified based on the accompanying temperature change as shown in Figure 3a,b, include:

(1) the initial linear, purely elastic loading (0)–(A14) accompanied by the temperature drop (0)–(A14 *);
(2) the nonlinear superelastic loading (A14)–(B14) related with the temperature growth (A14 *)–(B14 *);
(3) the transient stage (B14)–(C14) where the temperature starts growing fast (B14 *)–(C14 *);
(4) the plastic deformation (C14)–(D14) with a significant growth of temperature (C14 *)–(D14 *);
(5) the superelastic-like unloading (D14)–(E14) accompanied by a drop of temperature (D14 *)–(E14 *);
(6) the transient unloading (E14)–(F14) where the temperature starts decreasing slower (E14 *)–(F14 *);
(7) the elastic unloading (F14)–(G14), with a slight increase of temperature (F14 *)–(G14 *).

Stress *σ* accompanied by the related average temperature change Δ*T* vs. strain *ε* curves are separately shown, analyzed, and discussed in more detail in [55]. The determined temperature change was analyzed especially in the context of the superelastic-like deformation of the Gum Metal and the related unconventional mechanisms of the alloy deformation.

## 4. Background of Energy Balance in Solid Material during the Deformation Process

The energy phenomenon accompanying elastoplastic deformation is a challenge since it is strongly related to complex thermomechanical couplings. The relationships between the dissipation, the stored energy of cold work, and the material hardening should be taken into account. The data can be purchased and the analysis can be conducted on the macro, e.g., [4,5,10,11,12,13,14], meso, e.g., [8,23], and micro scales, e.g., [29]. In this paper, we consider the macroscopic approach to this issue. 

**Figure 4:**
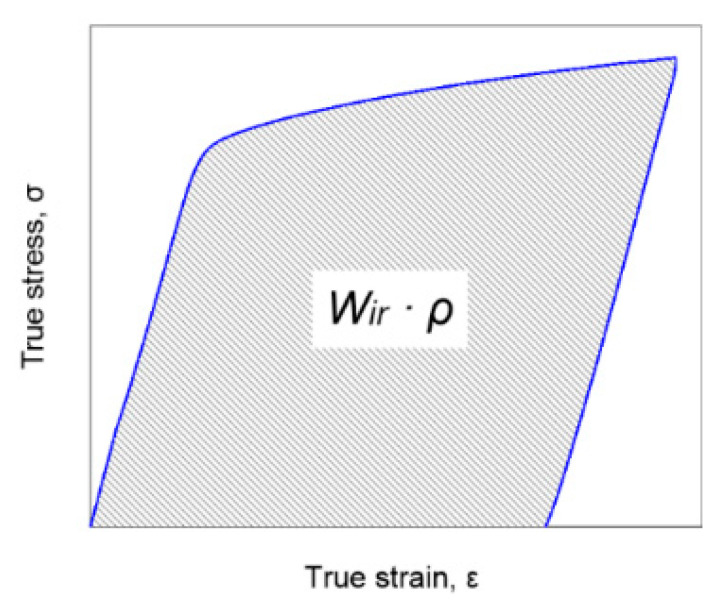
Schematic of methodology for determination of irrecoverable mechanical energy $W_{ir}$ expended on plastic deformation based on tensile load–unload stress vs. strain curve.

A schematic of the methodology is presented in Figure 4. In the case of elastic–plastic solids, the deformation work *W_ext_* is determined based on load–unload stress vs. strain curve and can be decomposed into a reversible part *W_rec_* and a complementary part *W_ir_*. 

The irrecoverable part $W_{ir}$ is determined as the area under the stress–strain curve taking into account the density ρ of the material (Figure 4).

The $W_{ir}$ can be decomposed into the dissipated part *W_dir_* and the stored energy of cold work *E_s_*.

(2) $$E_{s}=W_{ir}-W_{dir}$$

The stored energy $E_{s}\;\mathrm{represents}\;$ a change in the internal energy of the alloy (Equation (2)), usually related to defects of crystal lattice, and is an essential measure of cold-worked state [2,4,5,9].

In the initial studies on this topic by Taylor and Quinney, a part of plastic work stored in metal was believed to be about 10% of the entire plastic work [1]. However, further experimental studies and numerical simulation, discussed also in the Introduction of this paper, demonstrated that the ratio of the stored energy to the plastic work is usually larger and, first of all, is not constant during the material loading, but it depends on the stage of the deformation [2]. Therefore, the concept of the energy storage rate as a measure of energy conversion at each instant of the plastic deformation process was introduced. 

The rate of energy storage *Z* was defined as the irrecoverable plastic work $W_{ir}$ derivative of the stored energy *E_s_* and expressed by Equation (3):

(3) $$Z=\frac{dE_{s}}{dW_{ir}}$$

Analysis of the energy balance allows us to gain an additional, thermodynamic aspect insight into the nature of the deformation mechanisms involved in the loading process of a given material. 

## 5. Investigation of Energy Balance in Gum Metal Subjected to Consecutive Tensile Cycles

**Figure 5:**
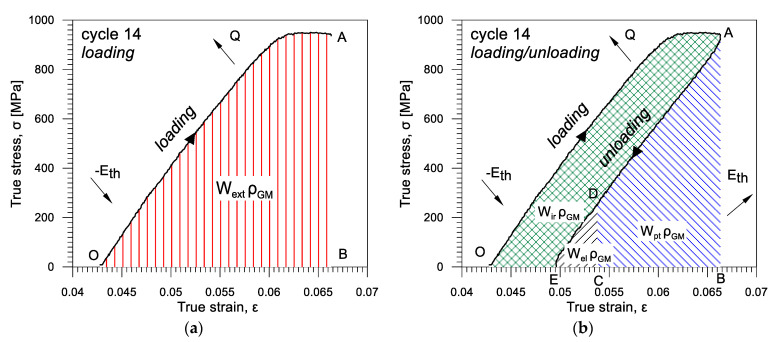
Methodology used for estimation of energy balance in Gum Metal during tension based on stress *σ* vs. strain *ε* loading–unloading curve: (**a**) the entire work $W_{ext}$; (**b**) work used for irrecoverable deformation $W_{ir}$ and work used for recoverable deformation $W_{rec}$ composed of work used for pure elastic deformation $W_{el}$ and superelastic-like $W_{pt}$.

In the case of materials demonstrating stress-induced transformation, e.g., shape memory or superelastic alloys, such as Gum Metal, the analysis of energy balance is more complex, since the exothermic forward and the endothermic reverse transformations should be taken into account as well. An approach to analyze energy balance during the Gum Metal deformation is demonstrated in Figure 5a,b, respectively. The loading–unloading cycle 14 (see Figure 2) was chosen for the analysis since, for such an advanced loading stage, various factors contribute to the process of the Gum Metal deformation; thus, all energy components can be considered.

The entire work needed to deform the alloy $W_{ext}$ from the beginning of the loading in point O until unloading in point A is equal to the area OAB, as presented in Figure 5a. $W_{ext}$ was divided into two parts used for recoverable $W_{rec}$ (area EAB, Figure 5b) and irrecoverable $W_{ir}$ deformation (area OAE, Figure 5b), as expressed by Equation (4):

(4) $$W_{ext}=W_{rec}+W_{ir}$$

In the case of Gum Metal, the work used for recoverable deformation $W_{rec}$ can be divided into the first part of work used for pure elastic deformation $W_{el}$ (area EDC) and the second part of work used for superelastic-like and mechanically recoverable deformation (caused by phase transformation of nanodomains) $W_{pt}$—area CDAB), as presented in Figure 5b and expressed by Equation (5):

(5) $$W_{rec}=W_{el}+W_{pt}$$

As mentioned above, the purely elastic deformation of Gum Metal, likely for any solids, is accompanied by a temperature drop during loading and a temperature increase during unloading, due to the thermoelastic effect [52,53,54]. The mechanically recoverable superelastic-like deformation caused by phase transformation of nanodomains, demonstrated by Kim, Miyazaki et al. in [49] is exothermic during the loading and endothermic during unloading, similarly as in the case of stress-induced transformation in TiNi SMA [58]. 

Thus, in this paper we try to demonstrate how the energy expended on plastic deformation *W_ext_*, as well as the particular energy components, including those associated with pure elasticity (thermoelastic effect) *E_th_* and the superelastic-like mechanically recoverable deformation *W_pt_*, change and develop during each of the 1–24 loading–unloading tension cycles.

The energy associated with the thermoelastic effect *E_th_* can be calculated using Equation (6) [59]:

(6) $$E_{th}=-\frac{\alpha T_{0}\Delta \sigma_{ik}}{\rho },$$

where

α is the coefficient of linear thermal expansion,
*T*_0_ is the absolute temperature of the specimen,
Δ*σ_ik_* is the stress tensor,
*ρ* is the density of the material.

The fraction of work used for irrecoverable deformation $W_{ir}$ means the work used for plastic deformation. $W_{ir}$ is equal to the heat-dissipated $W_{dir}$ and the energy stored $E_{s}$, as previously expressed by Equation (2). 

The entire heat-dissipated $W_{dir}$ in the process of deformation should include heat losses related to heat conductivity $W_{ind\;}$, convection $W_{conv}$, and radiation $W_{rad}$, as given in Equation (7).

(7) $$W_{dir}=Q+\;W_{ind\;}+\;W_{conv}+W_{rad}$$

The strain rate 10^−2^ s^−1^ used during the cyclic tension of Gum Metal was quite high, so, for the sake of simplicity, we can assume that the conditions were close to adiabatic and the heat losses were negligible. Therefore, we obtain Equation (8). The dissipated energy *W_dir_* is converted into heat and can be estimated by multiplying the specific heat of the material *c_p_* and the temperature change caused by the deformation Δ*T*.

(8) $$W_{dir}=Q=c_{p}\Delta T$$

where

*c_p_* is the specific heat of Gum Metal at constant pressure,
ΔT is the temperature change determined in the process of the plastic deformation.

The certain energies discussed above were calculated based on the mechanical Gum Metal characteristics by determining particular areas in the true stress vs. true strain curves and subsequently dividing the obtained values by the alloy density ρ. As the result, values of energies per mass unit measured in J/g were obtained.

Let us also assume for simplicity that the mechanically recoverable deformation of Gum Metal under cyclic tension occurs up to the same level of stress (730 MPa) for each loading–unloading cycle. However, one should be aware that certain microstructural changes induced to the alloy in the process of cyclic tension may affect this critical stress value.

Following the assumptions listed above, we obtained (Equation (9)),

(9) $$\Delta T=\Delta Tmax-\Delta T\left(730\;\mathrm{MPa}\right)$$

where $\Delta T\left(730\;\mathrm{MPa}\right)$ is the temperature change corresponding to the stress level at which the fully recoverable deformation of Gum Metal ends, as was experimentally found in [53]. 

This means that the entire work of $W_{ext}\;$ needed to deform the alloy is given by Equation (10):

(10) $$W_{ext}=E_{s}+Q+\;W_{el}+W_{pt}.$$

As a consequence, the stored energy can be expressed by Equation (11):

(11) $$E_{s}=W_{ext}-Q-W_{el}-W_{pt}.$$

The Gum Metal parameters used for estimation of energy balance in each loading–unloading cycle are given in Table 1. 

The density of Gum Metal *ρ* was measured using a commonly known Archimedes’ principle and equals 5.895 g/cm^3^. The thermal expansion coefficient *α* was taken from the literature [30]. The value of specific heat of Gum Metal *c_p_* was determined in a special experiment using a power-compensation differential scanning calorimeter Perkin-Elmer Pyris 1 DSC (Waltham, MA, USA) equipped with an Intracooler 2P cooling device. More technical details are presented in [55]. 

**Figure 6:**
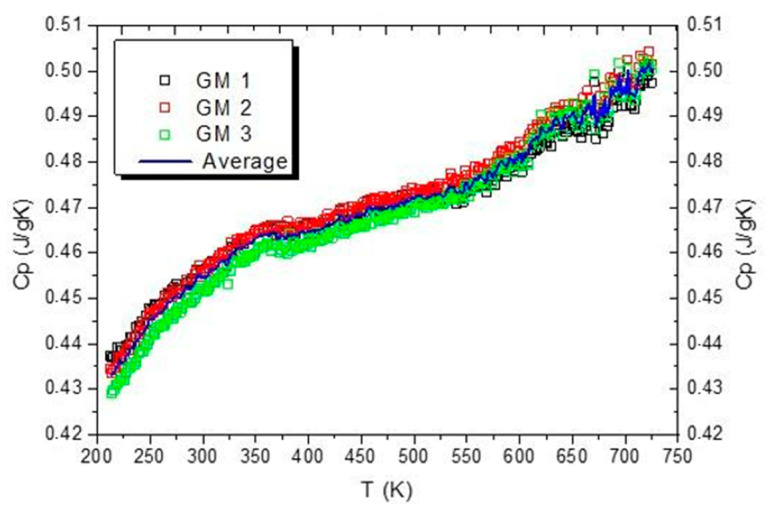
Specific heat vs. temperature curves for three samples of Gum Metal with an averaged curve.

Specific heat vs. temperature curves obtained for three samples of Gum Metal denoted by GM1, GM2, and GM3 with an averaged curve are presented in Figure 6. 

The values of Gum Metal specific heat determined based on the DSC measurements in the selected temperature range are between 0.43 J/gK and 0.50 J/gK with increasing temperature. At room temperature, the specific heat of Gum Metal is around 0.455 J/gK. This value was used for the energy analysis.

The values of stress Δ*σ* corresponding to the minimum temperature during the loading for Gum Metal subjected to cyclic tension at the strain rate 10^−2^ s^−1^ were derived from the obtained experimental data.

## 6. Estimation of Plastic and Dissipated Work and Stored Energy in Gum Metal Subjected to Subsequent Loading–Unloading Tensile Cycles—Discussion

**Figure 7:**
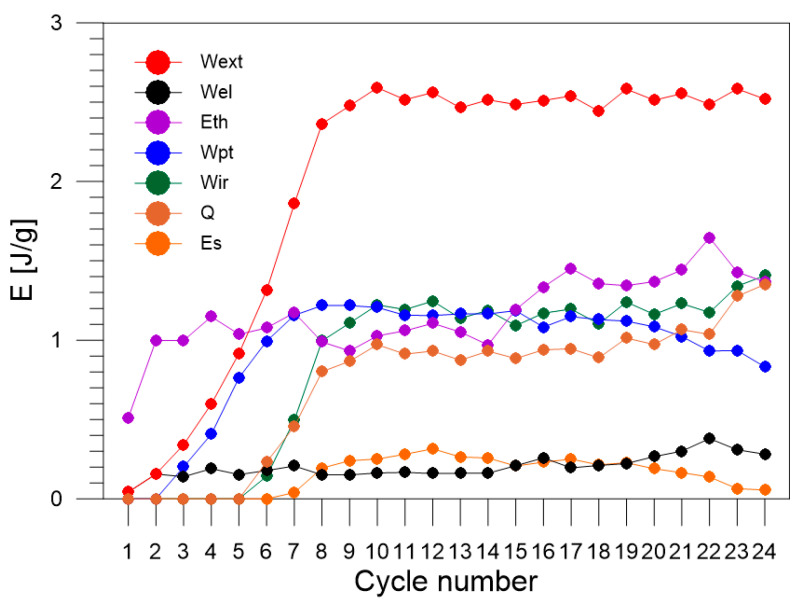
Values of energy balance components: $W_{ext}$, $W_{el}$, $W_{pt}$, $E_{th}$, Q, $W_{ir,}$ and $E_{s}$ estimated for Gum Metal in each of the 1–24 loading–unloading tension cycles.

**Figure 8:**
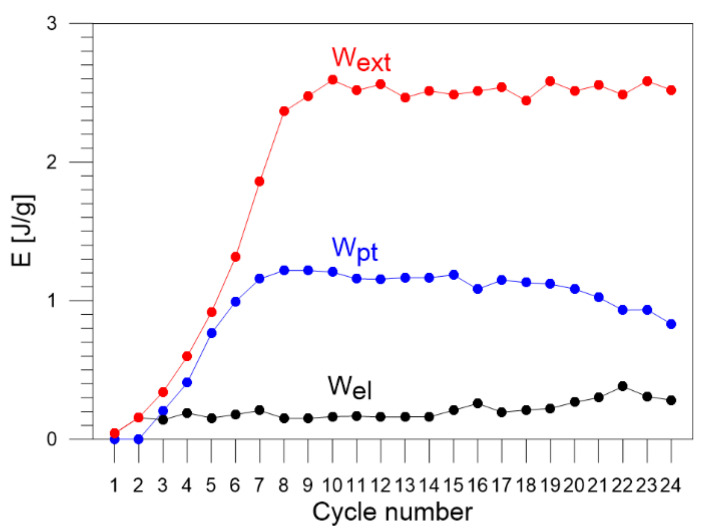
Estimated values of certain parts of energy: $W_{ext}$, $W_{el}$, and $W_{pt}$ for Gum Metal in each of the 1–24 subsequent loading–unloading tension cycles.

**Figure 9:**
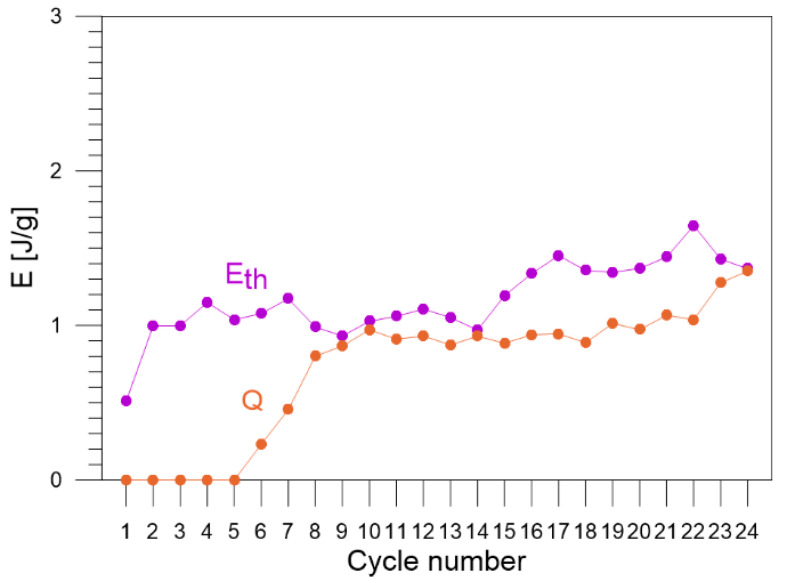
Values of $\;E_{th}$ and Q estimated for Gum Metal in each of the 1–24 loading–unloading tension cycles.

**Figure 10:**
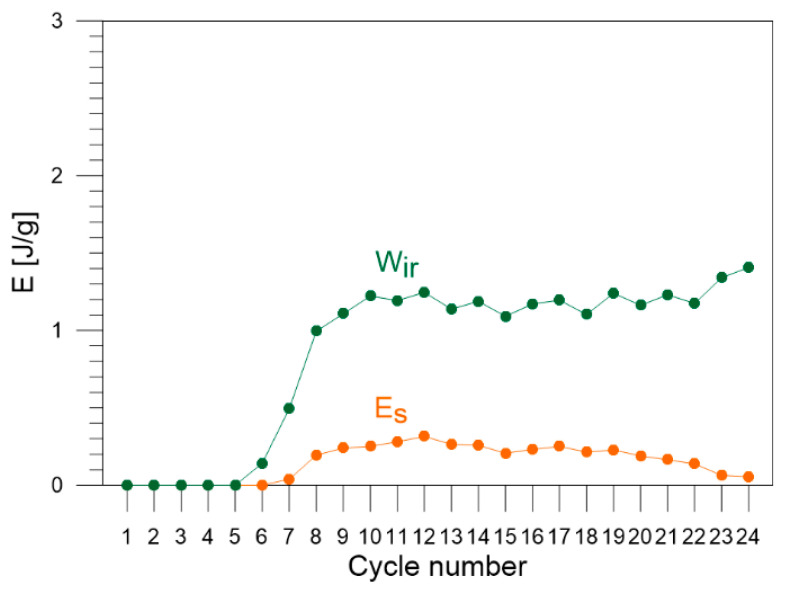
Values of certain parts of energy: $W_{ir}\;$ and $E_{s}$ estimated for Gum Metal in each of the 1–24 loading–unloading tension cycles.

The values of the energy estimated via the energy balance and the ratio Z (Equation (3)) for each loading–unloading cycle are listed in Table 2. The obtained energy components, $W_{ext}$, $W_{el}$, $W_{pt}$, $E_{th}$, Q, $W_{ir}$, $E_{s}$, estimated for each of the 1–24 loading–unloading cycles, are compared in Figure 7. In the next figures, selected energies are compared, i.e., estimated values of energy: $W_{ext}$, $W_{el}$, and $W_{pt}$ are shown in Figure 8; values of $E_{th}$ and Q in Figure 9; and values of energy $W_{ir}$ and $E_{s}$ in Figure 10, respectively. 

The values of the entire work needed to deform the Gum Metal $W_{ext}$ were calculated by determining particular areas in the true stress vs. true strain curves for each cycle of tensile loading. In the early tensile cycles 1–8 of Gum Metal loading–unloading process, $W_{ext}$ rises until reaching a value around 2.5 J/g and is pretty constant for the advanced cycles 9–24 (Figure 7 and Figure 8). It confirms similar mechanical behavior of the alloy in subsequent cycles which demonstrates a high quality of the Gum Metal, its stable properties, and low coefficient of the alloy hardening.

The value of part of the work used for elastic deformation of Gum Metal $W_{el}$ is very low in the cycle 1 which does not reach the total pure elastic strain and oscillates in the range 0.14–0.4 in subsequent cycles 2–24 (Figure 8). The discrepancy is affected by the inertia of the testing machine. 

The superelastic-like deformation of Gum Metal starts in cycle 3. The values of part of the work used for the superelastic-like mechanically recoverable deformation of Gum Metal caused by phase transformation of nanodomains $W_{pt}$ rise in cycles 3–8, remain constant in cycles 8–10 at the level of 1.2 J/g, and fall in the next cycles 10–24 in the range between 1.2 J/g and 0.9 J/g (Figure 8). 

The value of energy related to thermoelastic effect $E_{th}$ of Gum Metal equals 0.5 J/g in cycle 1 and subsequently reaches the values between 1 J/g and 1.6 J/g in cycles 2–24. The data reflect a slight hardening effect of the alloy in previous cycles. 

The values of heat generated by plastic deformation of Gum Metal *Q* are low in cycles 6 and 7 and, in subsequent cycles 8–24, they oscillate in the range between 0.8 J/g and 1.35 J/g, reaching the highest values in the last cycles 23–24. The obtained results confirm intense mechanisms of the specimen necking and damage (Figure 9).

The plastic deformation of Gum Metal initiates in cycle 6. The values of part of the work used for irrecoverable plastic deformation of Gum Metal $W_{ir}$ are low in cycles 6 and 7 where the range of plastic deformation is short. The values grow in subsequent cycles 8–24 and remain in the range between 0.9 J/g and 1.2 J/g. The highest values of $W_{ir}$ are obtained in cycles 23–24 in which the temperatures of the specimen are significant, due to various kinds of damage mechanisms dynamically developing just before the specimen rupture. 

The values of stored energy $E_{s}$ of Gum Metal estimated for each cycle of tensile loading are equal to zero in the early cycles 1–6 where there is no or only a little plastic deformation involved in the process of tension. In cycles 8–20, the values of $E_{s}$ remain in the range between 0.12 J/g and 0.25 J/g. Finally, the values of $E_{s}$ drop in cycles 21–24 (Figure 10), because the heat *Q* generated with increasing plastic deformation grows significantly due to the nucleation and development of damage mechanisms, i.e., macro shear bands, named giant faults [30,31,32,33]. 

**Figure 11:**
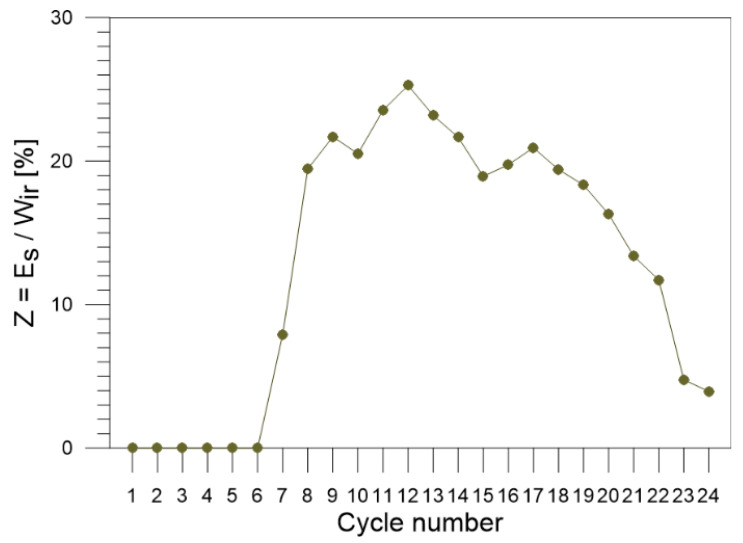
Values of the ratio $Z=\frac{E_{s}}{W_{ir}}$ calculated for Gum Metal for each of the 1–24 loading–unloading tension cycles.

The values of the ratio of the stored energy to the plastic work $Z=\frac{E_{s}}{W_{ir}}$ calculated for each of the 1–24 cycles of tensile loading of Gum Metal are presented in Figure 11.

The ratio of the stored energy to irreversible plastic work equals approximately 8% in cycle 7, grows to 20% in cycle 8, reaches the maximum of 26 % for cycle 12, and gradually decreases up to 3% in the last cycles, i.e., at the stage of the loading close to failure. Since the ratio *Z* is a measure of the energy conversion at each instant of the plastic deformation process, the diagram demonstrates in a reasonable way that the energy starts to store in the Gum Metal structure from cycle 7, increases fast for subsequent cycles, reaches its maximum exactly in the middle of the all cyclic loading processes, and decreases significantly at the higher cycles leading to the specimen necking and rupture (see Figure 2 and Figure 11). 

The high percentage value of the energy conversion for cycles 8–18 demonstrates that over 20% of the mechanical energy supplied to the Gum Metal, i.e., the irrecoverable plastic work $W_{ir}$ is converted into a change of its structure. 

At the stage close to the specimen rupture, the energy saturation is observed. It is demonstrated by the energy conversion values *Z* decreasing gradually to zero in the last tension cycles. Similar results were obtained for other alloys, e.g., [29]. 

## 7. Concluding Remarks

The estimation of macroscopic scale energy balance in Gum Metal was conducted for the consecutive loading–unloading 1–24 tensile cycles, until the specimen rupture. 

Particular energy components were determined experimentally in a macroscopic approach and analyzed using some thermodynamic relations related to the alloy deformation process. 

It was found that the irrecoverable plastic deformation $W_{ir}$ of Gum Metal initiates in cycle 6 at the strain value of approximately 0.016. The values remain low in cycles 6 and 7 since the range of plastic deformation is short. The values grow in subsequent cycles 8–24, and are between 0.9 J/g and 1.2 J/g. The highest values of $W_{ir}$ are obtained at higher number of cycles 23 and 24 in which the temperatures of the specimen rise significantly, caused by both advanced mechanisms of plastic deformation and damage mechanisms— namely macro shear bands, named giant faults—dynamically developing before the specimen rupture.

In the early cycles 1–6, the values of stored energy $E_{s}$ of Gum Metal estimated for each cycle of tensile loading are equal to 0, since there is no or only a little plastic deformation. In cycles 8–20, the values of $E_{s}$ are in the range between 0.12 J/g and 0.25 J/g. Finally, in cycles 21–24, the values of $E_{s}$ drop because the heat *Q* generated with increasing plastic deformation grows significantly due to the intense development of strong exothermic damage mechanisms.

The measure of the energy conversion at each instant of the plastic deformation process $Z=\frac{E_{s}}{W_{ir}}$ starts at cycle 6, equals approximately 8% for cycle 7 and 20% for cycle 8, increases to 26% for cycle 12 and, in general, remains in the range between 13% and 20% for cycles 8–18. In the last cycles, i.e., in the stage close to the Gum Metal specimen rupture, the values of the estimated ratio *Z* gradually decrease to zero.

The high percentage value of the energy conversion for cycles 8–18 demonstrates that over 20% of the mechanical energy supplied to the Gum Metal deformation, i.e., the irrecoverable plastic work $W_{ir}$, is converted into a change of its structure.

The gradual decreasing to zero values of the energy conversion, estimated in the last tension cycles, demonstrates the energy saturation observed at the stage close to the specimen rupture.

## Figures and Tables

**Table 1:** Parameters of Gum Metal used to estimate the energy balance.

Density *ρ* [g/cm^3^]	Specific Heat *c_p_* [J/gK]	Thermal Expansion Coefficient *α* [K^−1^]
5.895	0.455	8 × 10^−6^

**Table 2:** The obtained energy components: $W_{ext}$, $W_{el}$, $W_{pt}$, $E_{th}$, Q, $W_{ir}$, $E_{s},$ and the ratio Z, estimated for each of the 24 loading–unloading cycles of Gum Metal referred to the maximal stress value in each loading cycle.

		Values of Certain Energies of Gum Metal [J/g]							$\mathrm{Ratio}\;\frac{E_{s}}{W_{ir}}$
Cycle Number	Maximal Stress Value [MPa]	$W_{ext}$	$W_{el}$	$W_{pt}$	$W_{ir}$	$E_{th}$	Q	$E_{s}$	*Z* [%]
1	170.7	0.043	0.043	0	0	0.51	0	0	0
2	323.8	0.157	0.157	0	0	0.998	0	0	0
3	466.5	0.341	0.14	0.202	0	0.998	0	0	0
4	606.3	0.598	0.19	0.408	0	1.148	0	0	0
5	731.0	0.917	0.153	0.764	0	1.037	0	0	0
6	831.6	1.315	0.179	0.995	0.143	1.077	0.232	0	0
7	908.1	1.859	0.207	1.157	0.496	1.173	0.456	0.04	7.92
8	927.3	2.365	0.151	1.218	0.996	0.989	0.802	0.194	19.46
9	934.7	2.477	0.151	1.218	1.108	0.932	0.868	0.241	21.70
10	937.4	2.592	0.163	1.207	1.223	1.027	0.972	0.251	20.51
11	940.1	2.517	0.167	1.157	1.193	1.059	0.912	0.281	23.55
12	943.3	2.561	0.16	1.156	1.247	1.106	0.932	0.315	25.28
13	947.9	2.465	0.162	1.166	1.138	1.051	0.874	0.264	23.19
14	950.0	2.515	0.162	1.166	1.188	0.97	0.931	0.258	21.66
15	954.3	2.486	0.209	1.186	1.092	1.193	0.885	0.207	18.94
16	958.4	2.511	0.258	1.083	1.171	1.335	0.94	0.232	19.75
17	961.8	2.54	0.196	1.149	1.196	1.452	0.946	0.251	20.93
18	966.3	2.443	0.211	1.13	1.103	1.356	0.889	0.215	19.42
19	970.7	2.583	0.223	1.12	1.241	1.344	1.013	0.228	18.34
20	972.2	2.513	0.267	1.084	1.163	1.369	0.973	0.190	16.31
21	973.3	2.554	0.301	1.023	1.231	1.442	1.066	0.165	13.39
22	968.5	2.486	0.38	0.932	1.175	1.646	1.037	0.138	11.72
23	963.1	2.584	0.309	0.935	1.341	1.429	1.277	0.064	4.76
24	941.6	2.520	0.282	0.832	1.407	1.367	1.352	0.056	3.94

## Data Availability

The data will be available on reasonable request.
